# Oleoylethanolamide inhibits α-melanocyte stimulating hormone-stimulated melanogenesis via ERK, Akt and CREB signaling pathways in B16 melanoma cells

**DOI:** 10.18632/oncotarget.18097

**Published:** 2017-05-23

**Authors:** Juan Zhou, Tong Ren, Ying Li, Anran Cheng, Wanyi Xie, Lanxi Xu, Lu Peng, Jinbin Lin, Lianxiang Lian, Yong Diao, Xin Jin, Lichao Yang

**Affiliations:** ^1^ Department of Obstetrics and Gynecology, The First Affiliated Hospital of Xiamen University, Xiamen University, Xiamen, China; ^2^ Department of Pharmacy, Xiamen Medical College, Xiamen, China; ^3^ Xiamen Key Laboratory of Chiral Drugs, Medical College, Xiamen University, Xiamen, China; ^4^ School of Biomedical Sciences, Huaqiao University, Quanzhou, China

**Keywords:** oleoylethanolamide, melanin, tyrosinase, α-melanocyte stimulating hormone, B16 mouse melanoma cell

## Abstract

The present study aimed to examine the potential inhibitory activity of oleoylethanolamide (OEA) on α-melanocyte stimulating hormone (α-MSH)-stimulated melanogenesis and the molecular mechanism(s) involved in the process in B16 mouse melanoma cells. Our data demonstrated that OEA markedly inhibited melanin synthesis and tyrosinase activity in α-MSH-stimulated B16 cells. In addition, the expression of melanogenesis-related proteins, such as melanocortin-1 receptor (MC1R), microphthalmia-associated transcription factor (MITF), tyrosinase-related protein-1 (TRP-1) and tyrosinase, was suppressed in a concentration-dependent manner by OEA. In addition, OEA may suppress melanogenesis through a peroxisome proliferator-activated receptor α (PPARα)-independent pathway. Moreover, OEA activated ERK, Akt, p38 pathways and inhibits CREB pathway in α-MSH-stimulated B16 cells. The specific ERK inhibitor PD98059 partly blocked OEA-inhibited melanin synthesis and tyrosinase activity and partly abrogated the OEA-suppressed expression of melanogenic proteins. Furthermore, OEA presented remarkable inhibition on the body pigmentation in the zebrafish model system. Our findings demonstrated that OEA is an effective inhibitor of hyperpigmentation through activation of ERK, Akt and p38 pathways, inhibition of the CREB pathway, and subsequent down-regulation of MITF, TRP-1 and tyrosinase production.

## INTRODUCTION

Melanogenesis is a physiological process leading to the production of melanin pigment, which plays a vital role in the prevention of sun-induced skin injury and contributes to skin and hair color [1]. However, excessive generation of melanin results in hyperpigmentation, wrinkling, melasma, and other dermatological disorders [2]. Several known melanin synthesis inhibitors, including kojic acid, arbutin and many other natural products, are currently being utilized as cosmetic additives and have already been the focus of studies. However, the applicability of these chemicals might be limited given their adverse side effects. For example, kojic acid can lead to allergic dermatitis, and arbutin might carry carcinogenic risks. Therefore, finding new effective skin whitening products with low adverse effects would be valuable for cosmetic purposes and for clinical treatment of pigmentary disorders caused by melanin accumulation.

Melanogenesis is a complex process that is regulated by tyrosinase and tyrosinase related protein-1 and -2 (TRP-1 and TRP-2) [3]. Tyrosinase plays a pivotal role in altering melanin generation by the hydroxylation of tyrosine into dihydroxyphenylalanine (DOPA) followed by further oxidation of DOPA into DOPA quinone. Therefore, inhibition of tyrosinase is the most common approach to achieve skin hypopigmentation as it is the key enzyme that catalyzes the rate-limiting step of melanogenesis [4]. In addition, tyrosinase, TRP-1 and TRP-2 are transcriptionally regulated by a crucial transcription factor in melanocytes, namely, microphthalmia-associated transcription factor (MITF) [5]. Skin pigmentation is regulated by a variety of extrinsic and intrinsic factors [6]. In particular, extracellular signal-regulated kinase (ERK) negatively regulates melanogenesis in melanocytes and melanoma cells [7, 8] and is also an effective modulator of the activation of MITF, thereby leading to the regulation of melanogenesis.

Peroxisome proliferator-activated receptors (PPARs) are a group of nuclear receptor proteins that are associated with various skin diseases, particularly those involving inflammation, epidermal hyperproliferation, and differentiation [9, 10]. PPARα agonists exhibit the potential therapeutic application for the treatment of pigmentary disorders, including vitiligo, melasma and postinflammatory hyperpigmentation [11]. In clinical trials, fewer patients treated with gemfibrozil, a PPARα agonist, were diagnosed with melanoma compared with the control group [12]. Fenofibrate, another PPARα agonist, suppresses melanogenesis through activation of the p38 mitogen-activated protein kinase pathway [13]. Previous studies have demonstrated that oleoylethanolamide (OEA), the potent endogenous ligand of PPARα, possessed antihyperlipidemia and weight reduction properties [14–16]. Moreover, our previous study showed that OEA exhibited potent neuroprotective and anti-inflammatory effects [17–19]. Although the positive effects of other PPARα agonists on inhibiting melanogenesis have been verified, little is known about the anti-melanogenic activity of OEA.

In the present study, we investigated the effects of OEA on the regulation of melanogenesis in α-MSH-treated B16 melanoma cells and zebrafish. In addition, we tested whether the PPARα is a potentially interesting target for OEA in inhibiting melanogenesis. Moreover, the involvement of the molecular mechanism(s) in OEA effects on melanogenesis was also investigated through the inhibition experiments.

## RESULTS

### OEA inhibits melanin synthesis and tyrosinase activity in α-MSH-stimulated B16 cells at non-cytotoxic dosages

**Figure 1:**
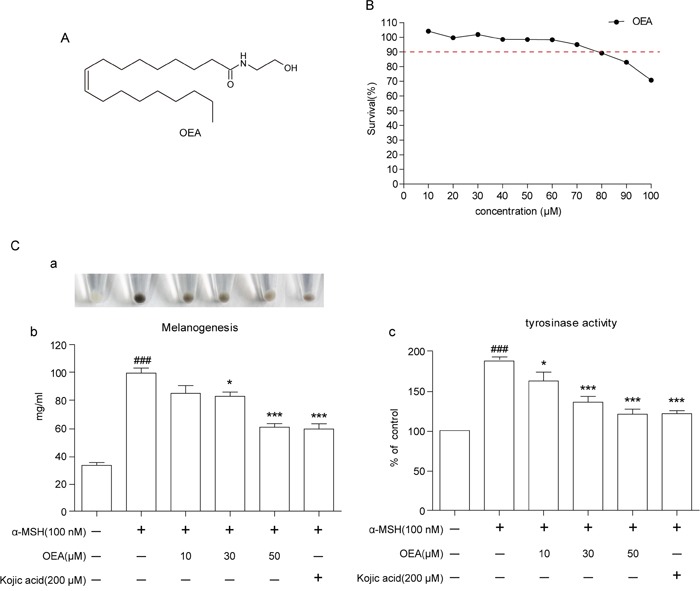
Effect of OEA on cellular melanin synthesis and tyrosinase activity in α-MSH-stimulated B16 cells **(A)** The chemical structure of OEA. **(B)** Cells were treated with various concentrations of OEA for 72 h. Cell viability was determined by MTT assay. **(C)** Relative cellular melanin content and tyrosinase activity were measured at 72 h after treatment. Cells were exposed to 100 nM α-MSH in the presence of 10 to 50 μM OEA or 200 μM kojic acid. The percentage values of the treated cells are expressed relative to that in control cells. Kojic acid was used as a positive control. Data are reported as the mean ± SEM of three independent experiments performed in triplicate (n=3). ^###^*P*<0.001 vs. control group; *P<0.05, ***P<0.001 vs. α-MSH-stimulated group.

To avoid the possibility that inhibition of melanogenesis is due to cytotoxicity, we first performed MTT assays to determine whether OEA is cytotoxic to B16 cells. B16 cells were treated with different concentrations of OEA (10-100 μM) for 72 h. The results showed that treatment of OEA caused mild cytotoxicity at the dosage of 80 μM (Figure 1B). Thus, 10 to 50 μM doses were chosen to determine the effects of OEA on melanogenesis and tyrosinase activity. The results showed that OEA reduced melanin content and tyrosinase activity in a dose-dependent manner in α-MSH-stimulated B16 cells. At 10, 30 and 50 μM OEA, the melanin content was decreased by 11.99%, 13.69% and 32.00%, respectively (Figure 1C–b), and tyrosinase activity was decreased by 15.23%, 28.52% and 32.98%, respectively (Figure 1C–c). Kojic acid, a well-known melanogenesis inhibitor, was used as a positive control. Moreover, the inhibition effect of kojic acid 200 μM was similar to 50 μM OEA. Meanwhile, we did not observe that OEA inhibits melanin synthesis and tyrosinase activity in normal B16 cells (Supplementary Figure 2).

### OEA decreases MC1R, MITF, TRP-1 and tyrosinase protein expression in α-MSH-stimulated B16 cells

**Figure 2:**
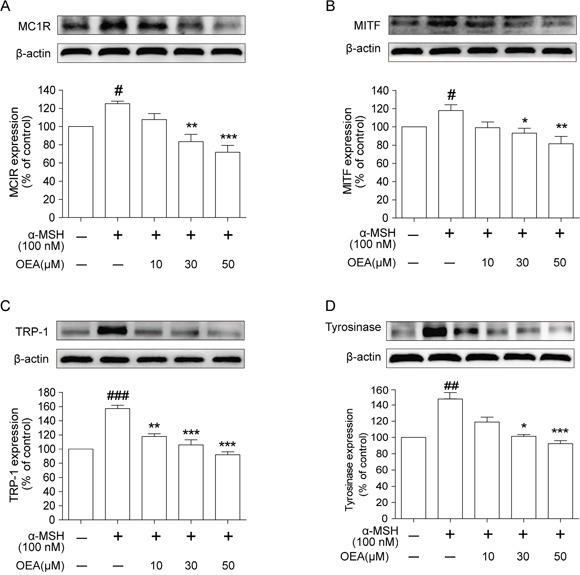
Effect of OEA on the expression of melanogenesis-related proteins in α-MSH-stimulated B16 cells Cells were exposed to 100 nM α-MSH in the presence of 10 to 50 μM OEA for 72 h. **(A)** MC1R, **(B)** MITF, **(C)** TRP-1 and **(D)** tyrosinase protein levels were examined by Western blot. The data are presented as percentages compared with the control group (set to 100%) and represented as the means ± SEM of three separate experiments performed in duplicate (n=3). ^#^*P*<0.05, ^##^*P*<0.01, ^###^*P*<0.001 vs. control group; *P<0.05, *P<0.01, ***P<0.001 vs. α-MSH-stimulated group.

To determine whether the suppressive activity of OEA is associated with expression levels of melanogenesis-related proteins, such as MCR1, MITF, tyrosinase-related protein-1 (TRP-1), and tyrosinase, cells were exposed to α-MSH with or without OEA treatment, and protein extracts were then subjected to Western blot analysis. As shown in Figure 2, MCR1, MITF, TRP-1 and tyrosinase protein expression levels in B16 cells treated with various concentrations of OEA were reduced in a dose-dependent manner compared with control. The data suggest that the inhibition of melanogenesis by OEA is associated with the down-regulation of MCR1/MITF-tyrosinase signaling pathways.

### PPARα signaling does not mediate the effect of OEA on melanogenic activity

**Figure 3:**
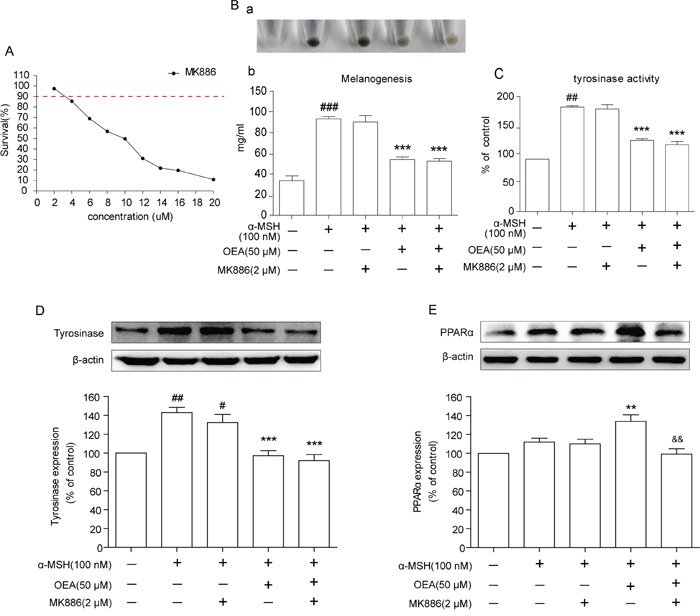
Effects of OEA on the PPARα signaling in α-MSH-stimulated B16 cells **(A)** Cells were treated with various concentrations of MK886 for 72 h. B16 cells were pretreated with DMSO only or MK886 (2 μmol/L) for 1 h followed by the addition of 100 nM α-MSH in the absence or in the presence of 50 μM OEA. **(B)** Relative melanin content and **(C)** tyrosinase activity were measured, and **(D** and **E)** the cell lysates were isolated through Western blot at 72 h after OEA treatment. The data are presented as percentages compared with the control group (set to 100%) and represented as the means ± SEM of three separate experiments performed in duplicate (n=3). ^#^*P*<0.05, ^##^*P*<0.01, ^###^*P*<0.001 vs. control group; *P<0.01, ***P<0.001 vs. α-MSH-stimulated group; ^&&^*P*<0.01 vs. α-MSH+OEA group.

To elucidate the mechanism of OEA on melanin production, we sought to determine whether the effects of OEA are mediated through activation of the PPARα receptor. We also examined the effect of MK886, an inhibitor of PPARα, on melanin levels, tyrosinase activity and tyrosinase expression. We first tested the non-cytotoxic concentration in B16 melanoma cells using the MTT assay, and 2 μM MK886 was determined as the proper concentration for further study (Figure 3A). The suppression of melanin content, tyrosinase activity and tyrosinase expression by OEA was not altered by MK886 pretreatment (Figure 3B–b, 3C and 3D). In contrast, MK886 could override the OEA-induced repression of PPARα (Figure 3E). Thus, these results imply that the biological effects of OEA are not dependent on PPARα signaling.

### OEA inhibits melanogenesis in α-MSH-stimulated B16 cells through activating the ERK pathway

**Figure 4:**
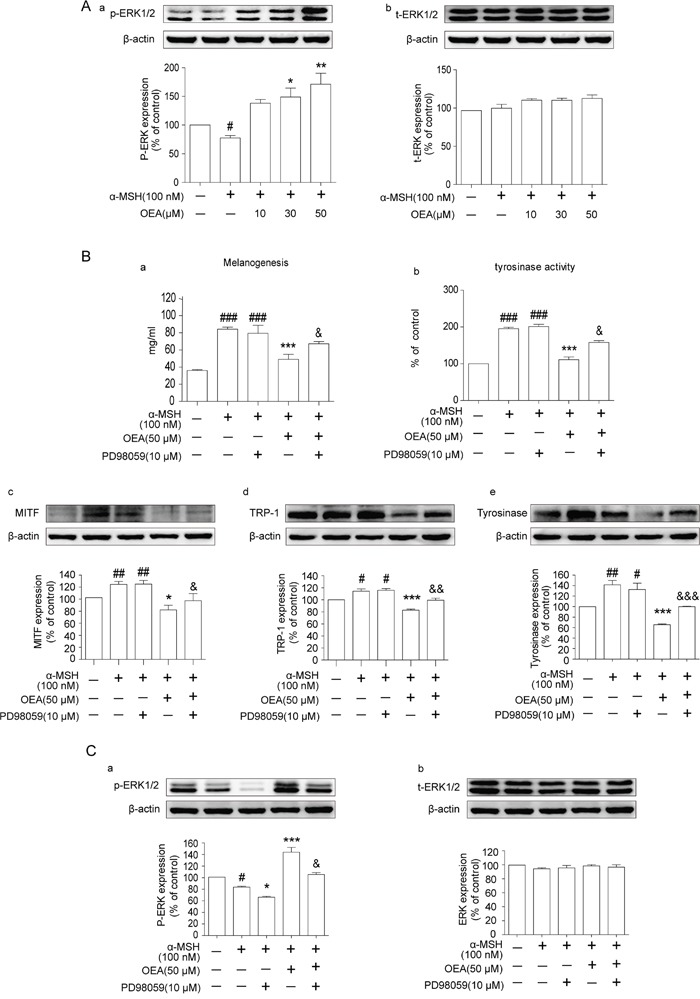
Effects of OEA on the ERK signaling pathway in α-MSH-stimulated B16 cells B16 cells were pretreated with DMSO only or PD98059 (10 μmol/L) for 1 h followed by the addition of 100 nM α-MSH in the absence or in the presence of 50 μM OEA. **(B-a)** Relative melanin content and **(B-b)** tyrosinase activity were measured, and **(A, B-c, B-d, B-e**, and **C)** the cell lysates were isolated through Western blot at 72 h after OEA treatment. The data are presented as percentages compared with the control group (set to 100%) and represented as the means ± SEM of three separate experiments performed in duplicate (n=3). ^#^*P*<0.05, ^##^*P*<0.01, ^###^*P*<0.001 vs. control group; *P<0.05, *P<0.01, ***P<0.001 vs. α-MSH-stimulated group; ^&^*P*<0.05, ^&&^*P*<0.01, ^&&&^*P*<0.001 vs. α-MSH+OEA group.

ERK activation induces the degradation of MITF, which subsequently decreases the expression of tyrosinase [20]. To further understand the inhibition of OEA on melanogenesis, we examined ERK and p-ERK protein levels in α-MSH stimulated B16 cells. Our results demonstrated that α-MSH stimulation reduced the levels of p-ERK1/2 in B16 cells. Therefore, this result indicates that ERK1/2 is involved in melanogenesis in B16 cells. However, OEA significantly increased p-ERK1/2 levels in a dose-dependent manner (Figure 4A–a). Total ERK1/2 levels exhibited no difference among the groups (Figure 4A–b). Thus, our results indicated that ERK1/2 signaling is associated with the effects of OEA on melanogenesis alteration in B16 cells.

To further confirm the role of the ERK pathway on the OEA-induced anti-melanogenic effect, we used a specific inhibitor of ERK, PD98059, which blocks ERK signaling. Cells were pre-treated with PD98059 in the presence of α-MSH and OEA, and then we measured melanin levels and tyrosinase activity. The results indicated that the synergistic effect of α-MSH and OEA on melanin production and tyrosinase activity was partially offset by PD98059 treatment (Figure 4B–a and 4B–b). Additionally, to confirm our results, we further examined the effect of PD98059 on the expression of MITF, TRP-1 and tyrosinase in the presence of α-MSH or OEA. PD98059 partially restored OEA-induced MITF, TRP-1, and tyrosinase down-regulation in α-MSH-stimulated B16 cells (Figure 4B–c, 4B-d and 4B-e). We next examined whether PD98059 inhibits the ERK pathway in α-MSH-stimulated B16 cells and found that PD98059 does inhibit ERK activation in OEA-treated B16 cells (Figure 4C). Therefore, our results imply that OEA-induced inhibition of melanogenesis through activating the ERK signaling pathways.

### Akt, CREB and p38 signaling pathways are involved in the suppressive effect of OEA on melanogenesis

**Figure 5:**
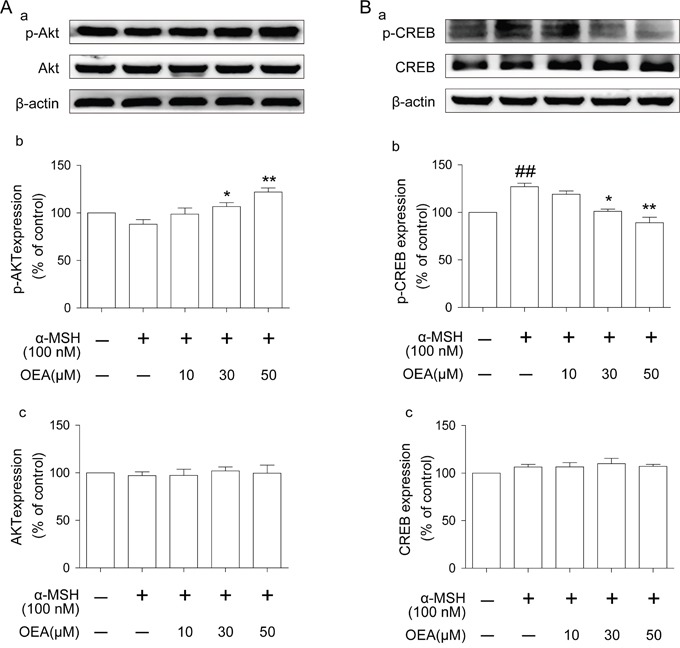
Effect of OEA on Akt and CREB signaling pathways in α-MSH-stimulated cells **(A-a** and **B-a)** The protein levels of p-Akt, Akt, p-CREB, and CREB were examined by Western blot. **(A-b, A-c, B-b**, and **B-c)** The data are presented as percentages compared with the control group (set to 100%) and represented as the means ± SEM of three separate experiments performed in duplicate (n=3). ^##^*P*<0.01 vs. control group; *P<0.05, *P<0.01 vs. α-MSH-stimulated group.

To further elucidate the mechanisms underlying the anti-melanogenic effect of OEA, we examined whether OEA influences Akt, CREB and/or p38 activation. As shown in Figure 5 and Supplementary Figure 1, OEA induces Akt and p38 phosphorylation but inhibits CREB phosphorylation in a dose-dependent manner. Total Akt, CREB and p38 levels did not differ among the groups. Thus, these results imply that OEA-induced inhibition of melanogenesis occurs through Akt, CREB and p38 signaling pathways.

### OEA suppresses melanogenesis in zebrafish

**Figure 6:**
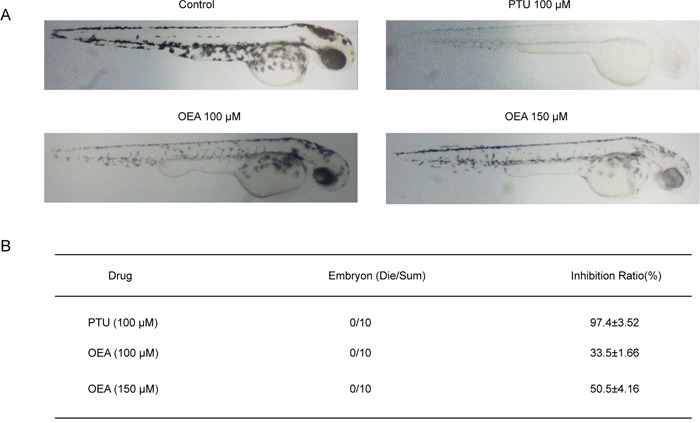
Effects of OEA on melanogenesis in zebrafish **(A)** Representative photographs of zebrafish. Synchronized embryos were treated with melanogenic inhibitors at the indicated concentrations. The compounds were dissolved in 0.05% DMSO and then added to the embryo medium. The effects on the pigmentation of zebrafish were observed under the stereomicroscope. **(B)** Melanin pigment. Approximately 10 embryos were collected, and the stereomicroscope was used to observe the effects on the pigmentation of zebrafish at 48 hours post fertilization. Images capturing and pixel measurement analysis were performed by ImageJ and Photoshop.

Zebrafish has melanin pigments on the surface, allowing simple observation of the pigmentation process without complicated experimental procedures. Therefore, we further tested the effects of OEA on zebrafish pigmentation. We used PTU as a positive control, which is a sulfur-containing tyrosinase inhibitor that is used widely in zebrafish research [21]. As shown in Figure 5A, 150 μM OEA produced remarkable inhibition on body pigmentation. Compared with untreated embryos, 100 and 150 μM OEA exposure resulted in 33.5±1.66% and 50.5±4.16% reduction in melanogenesis, respectively. In addition, 100 μM PTU and 100 μM and 150 μM OEA showed no significant effect on embryo survival rate (Figure 6B). When assessing morphological malformations, OEA did not exhibit conspicuous adverse effects (data not shown).

## DISCUSSION

A previous report demonstrated that OEA is a potent endogenous ligand of PPARα [14]. The administration of OEA, as a pharmacological drug, modulates feeding, lipid metabolism, glucose homeostasis, and anti-atherosclerotic functions through activation of the PPARα signaling pathway [14, 16, 22]. Furthermore, our previous study showed that OEA exhibited potent neuroprotective and anti-inflammatory effects [17–19]. However, despite numerous published results, the effect of OEA on melanogenesis has not been reported. In the present study, we investigated the effect of OEA on melanin synthesis and the molecular mechanism(s) involved in the process in B16 mouse melanoma cells.

Melanin levels directly correlate with the protein levels of tyrosinase and the activity of tyrosinase [23]. We first investigated whether OEA could inhibit melanin synthesis in the presence of α-MSH. Kojic acid is a kind of melanoma specific inhibitor, it changes the three-dimensional structure of tyrosinase and prevents tyrosinase activation, and then inhibits the formation of melanin [24]. Therefore, kojic acid was used as a positive control in the *vitro* study. We found that OEA inhibited melanin synthesis and tyrosinase activity in a concentration-dependent manner without cytotoxicity (10–50 μM) in B16 cells and that the extent of inhibition was comparable between treatment with OEA (50 μM) and with kojic acid (200 μM). These results demonstrate that OEA inhibits melanin synthesis and down-regulates tyrosinase activity in α-MSH-stimulated B16 cells. In general, through binding to MC1R, α-MSH potently induces MITF expression, which controls pigmentation by regulating the expression of melanogenic enzymes, such as TRP-1, TRP-2, and tyrosinase, that increase melanin synthesis [25]. Therefore, it is important to verify the inhibitory effect of OEA on melanogenesis by assessing the down-regulation of MITF and TRP expression. Our results indicate that OEA significantly reduces MC1R, MITF, TRP-1 and tyrosinase protein levels in α-MSH-stimulated B16 cells. These data suggest that OEA does not directly inhibit tyrosinase activity and that OEA's inhibitory effect results in MITF and TRPs down-regulation in B16 cells.

PPARα is a ligand-activated transcription factor that is involved in various skin diseases, such as psoriasis, acne, atopic dermatitis, scleroderma and melanoma [26]. PPARα is a major research target for the understanding and treatment of numerous skin pathologies, including hyperproliferative and inflammatory diseases. To evaluate whether the suppressive melanogenesis of OEA was induced through PPARα, we examined the effect of MK886, an inhibitor of PPARα on melanin levels, tyrosinase activity and tyrosinase expression. Our results revealed that OEA reduces melanin content and tyrosinase activity, and these phenomena were not reversed by the PPARα antagonist MK886. In addition, OEA increased PPARα protein expression in α-MSH-stimulated B16 cells, but this effect was almost abrogated by MK886 treatment. Our results imply that the biological effects of OEA are not dependent on PPARα signaling. Moreover, a previous study revealed that fenofibrate, an another PPARα agonist, inhibited melanin synthesis through a PPARα-independent pathway [13]. Therefore, these results suggest that the PPARα pathway may not be involved in the inhibition of PPARα agonists on melanogenesis.

The ERK pathway is involved in the regulation of melanin synthesis. The activation of ERK leads to phosphorylation of MITF at serine 73, resulting in its ubiquitination and degradation [27]. In addition, numerous natural and synthetic agents inhibit melanogenesis through activation of the ERK pathway [7, 27]. Therefore, we examined the influence of OEA treatment on the activation of ERK to further understand the molecular mechanisms involved in the pigmentation property of OEA. The data showed that ERK phosphorylation was significantly enhanced after OEA treatment. This finding suggested that OEA-induced anti-melanogenesis in B16 cells is related to the ERK-mediated pathway. To further determine whether OEA suppresses melanin synthesis and tyrosinase activity through the ERK pathway, we treated B16 cells with the ERK-specific inhibitor PD98059. We found that the suppressive effects of OEA on melanin synthesis and tyrosinase activity were partially diminished by prior inhibition of the ERK pathway. Moreover, we also confirmed that OEA-induced MITF, TRP-1 and tyrosinase protein down-regulation was also partially reversed by PD98059 in α-MSH-stimulated B16 cells. These results suggests that OEA inhibits melanin synthesis and tyrosinase activity through ERK signaling pathway-mediated suppression of MITF, TRP-1 and tyrosinase in α-MSH-stimulated B16 cells. Therefore, our results further confirmed that the ERK signaling pathway plays an essential role in the regulation of melanogenesis.

Our results also indicate that there may be other pathway(s) involved in the inhibition of melanin synthesis by OEA because the anti-melanogenic effect of OEA was not completely abrogated by inhibition of the ERK pathway. Transcriptional regulation of tyrosinase expression is mainly dependent on MITF, which is up-regulated by CREB and down-regulated by PI3K/Akt. Activation of the PI3K/Akt pathway reduces tyrosinase transcription and melanogenesis in B16F10 cells [28]. The p38 mitogen-activated protein kinase (MAPK) pathway is also involved in the regulation of melanogenesis [29]. CREB is an important regulator of MITF. Once phosphorylated, CREB up-regulates MITF, which binds to M-box and E-box motifs in the promoter of target genes including melanogenesis for transcriptional up-regulation of the key enzyme in melanin production [30]. Therefore, we further examined whether OEA activates/inhibites Akt, CREB and p38 pathways. In the present study, our results showed that OEA significantly increased the activity of Akt and p38 but inhibited CREB activation. These results suggest that OEA-induced Akt, CREB and p38 signalings may also play an important role in melanogenesis regulation in B16 cells.

Furthermore, we tested the effects of OEA on the pigmentation of zebrafish. Zebrafish is a highly advantageous vertebrate model organism given its similar gene sequences and organ systems to humans. In the present study, the results showed that OEA decreased body pigmentation. OEA was determined to effectively suppress the production of melanin *in vitro* and inhibit body pigmentation of zebrafish *in vivo*. Therefore, one interesting finding of our study is the consensus between the results obtained from *in vitro* and *in vivo* experiments.

**Figure 7:**
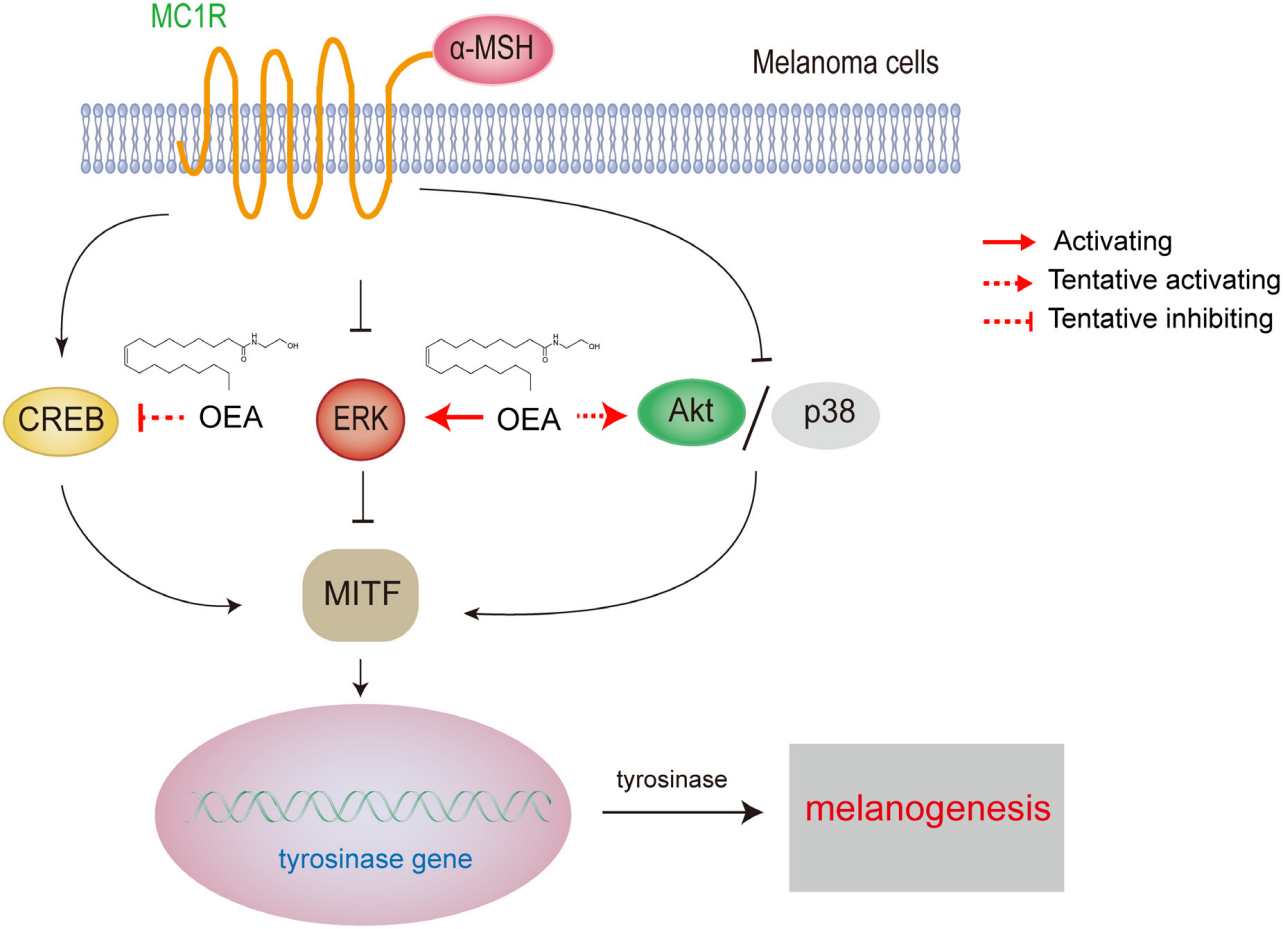
The mechanism of OEA inhibition of α-melanocyte stimulating hormone-stimulated melanogenesis in B16 melanoma cells

In summary, our findings demonstrated that OEA inhibits melanin synthesis and tyrosinase activity through ERK pathway-mediated suppression of MITF and TRP-1 in α-MSH-stimulated B16 cells. In addition, OEA-induced anti-melanogenesis may also be associated with Akt, CREB and p38 signaling pathways regulation (Figure 7). Moreover, OEA exerts an inhibitory effect on melanogenesis through a PPARα-independent pathway. Therefore, OEA may be a useful therapeutic agent for use in the treatment of hyperpigmentation and may be an effective component in whitening and lightening cosmetics.

## MATERIALS AND METHODS

### Materials

Dulbecco's modified Eagle's medium (DMEM), fetal bovine serum (FBS), penicillin and streptomycin were purchased from Gibco BRL (Carlsbad, CA, USA). OEA, kojic acid, MK886, L-DOPA, dimethyl sulfoxide (DMSO), 3-(4,5-dimethylthiazol-2-yl)-2,5-diphenyltetrazolium bromide (MTT), α-melanocyte-stimulating hormone (α-MSH), PD98059 and PTU were purchased from Sigma-Aldrich Co. (St Louis, MO, USA). Antibodies against MC1R (1:1000, Cell Signaling Technology, Boston, USA), MITF (1:1000, Cell Signaling Technology), TRP-1 (1:1000, Cell Signaling Technology), Tyrosinase (1:1000, Cell Signaling Technology), PPARα (1:1000, Abcam, Cambridge, UK), total-ERK (1:1000, Cell Signaling Technology), phospho-ERK (Thr202/Thr204) (1:1000, Cell Signaling Technology), total-Akt (1:1000, Cell Signaling Technology), phospho-Akt (Ser473) (1:1000, Cell Signaling Technology), total-CREB (1:1000, Cell Signaling Technology), phospho-CREB (Ser133) (1:1000, Cell Signaling Technology), total-p38 (1:1000, Cell Signaling Technology), phospho-p38 (1:1000, Cell Signaling Technology) and mouse monoclonal anti-β-actin (1:10000, Sigma) were obtained from the indicated manufacturers. OEA, kojic acid, MK886, PD98059 and PTU were dissolved in dimethyl sulfoxide (DMSO), and the final DMSO concentration was less than 0.05%. L-DOPA was dissolved in phosphate buffered saline (PBS).

### Cell culture

Murine B16 melanoma cells were obtained from the Type Culture Collection of the Chinese Academy of Sciences, Shanghai, China. Cells were grown in DMEM supplemented with 10% heat-inactivated FBS and 1% penicillin/streptomycin at 37°C in a humidified atmosphere of 5% CO_2_. For experiments, melanocytes were used from passages 2 to 10.

### Cell viability assay

To examine the effect of OEA on cell viability, the MTT assay was performed according to the manufacturer's instructions. Briefly, B16 cells were seeded in 96-well plates (5 × 10^3^ cells/well) and allowed to adhere at 37°C for 24 h. Cells were then treated with various concentrations of OEA for 72 h. After treatment, 100 μl of MTT solution (5 mg/ml in PBS) was added to each well, and cells were incubated at 37°C for 4 h. Following medium removal, 100 μl of DMSO was added to each well, and plates were gently shaken for 5 min. Optical absorbance was determined at 490 nm with a microplate spectrophotometer (BD Bioscience, USA). Absorbance of cells without treatment was regarded as 100% of cell survival. Each treatment was performed in triplicate, and each experiment was repeated thrice.

### Measurement of cellular melanin contents

The effect of OEA on α-MSH-induced melanogenesis in B16 cells was investigated according to a previously published protocol with slight modifications [31]. Briefly, B16 cells were cultured at 1×10^5^ cells/ml in 6-well plates as described above for 24 h. Then, the cells were treated with α-MSH (100 nM) in the absence and presence of different concentrations of OEA (10, 30, and 50 μM) or kojic acid (200 μM) for an additional 72 h. After treatment, cells were washed in ice-cold PBS and dissolved in 1 ml of 1 M NaOH containing 10% DMSO at 90°C for 60 min. Then, absorbance was measured at 405 nm using the SpectraMax M5 microplate reader (Molecular Devices Corp., Sunnyvale, CA). To measure the amount of melanin in the experiment, the rate of inhibition in the treatment groups was calculated by the absorbance of known concentrations of synthetic melanin, correcting for the total amounts of protein that are present in the supernatants of cell lysates [32].

### Measurement of cellular tyrosinase activity

Tyrosinase activity was estimated by measuring the rate of production of dopachrome from L-DOPA. B16 cells were treated as described above. Then, the cells were treated with α-MSH (100 nM) in the absence and presence of different concentrations of OEA (10, 30, and 50 μM) or kojic acid (200 μM) for an additional 72 h. After treatment, cells were washed twice with PBS and homogenized with 150 μl ice-cold PBS containing 1% (w/v) Triton X-100 (Sigma) and 0.1 mM phenylmethanesulfonylfluoride (PMSF) by freezing and thawing. The lysates were clarified by centrifugation at 13,000 r/min for 20 min, and then supernatants were collected. Afterward, the cellular extracts (90 μl) were transferred into a freshly prepared 10 μl L-DOPA solution (0.25% in PBS) in a well of a 96-well plate and incubated in the dark for 1 h at 37°C. After incubation, the generated dopachrome was monitored by an ELISA reader at the absorbance of 405 nm using the SpectraMax M5 microplate reader (Molecular Devices Corp., Sunnyvale, CA). Protein quantification of each lysate was performed using the BCA protein assay kit. For data analysis, the activity was calculated in units divided by the amount of protein measured.

### Western blot analysis

The protein samples (30 μg) were separated on 10% SDS-PAGE gels and transferred onto polyvinylidene difluoride membranes (Millipore, Billerica, MA, USA). The membranes were blocked with 5% BSA in Tris-buffered saline containing 0.1% Tween 20 and incubated with specific antibodies. The values were normalized to the β-actin intensity levels. The immunoblots were detected with enhanced chemiluminescence reaction reagents, and the images were captured with Image Station 4000R (Rochester, New York, USA).

### Effects on zebrafish embryo pigmentation

In addition to estimating the effects of OEA on melanogenesis *in vivo*, we further evaluate its anti-pigmentation ability in zebrafish according to the previous method with slight modification [33]. Embryos were obtained from natural spawning (School of life sciences, Xiamen University), which was induced in the morning by turning on the light. Briefly, 10 to 15 zebrafish embryos were transferred into 24-well plates, which were maintained in 1 ml embryo medium solution with a 14/10 hour light/dark cycle at 28.5°C. OEA was dissolved in DMSO and added to the embryo medium at the final concentration of 100 and 150 μM from 9 to 48 hours post-fertilization (h.p.f.) (39 hours exposure). In addition, 100 μM PTU considered as a standard positive control. To ensure drug stability, the dressing was changed every 12 h during this period. A stereomicroscope was employed to observe the effects on the pigmentation of zebrafish at 48 h.p.f. Image capturing and pixel measurement analysis were performed using ImageJ and Photoshop. The quantification of pigmentation data was calculated as the percentage change in comparison with the control group, which was considered as 100%.

### Statistical analysis

Results are expressed as the means ± standard error (SEM). Data were analyzed by one-way analysis of variance (ANOVA) of the differences within treatments followed by Tukey's post hoc test (Prism 5 for Windows, GraphPad Software Inc., USA). *P* < 0.05 was considered statistically significant.

## SUPPLEMENTARY MATERIALS FIGURES


