# Supplementary material for: Oleoylethanolamide inhibits α-melanocyte stimulating hormone-stimulated melanogenesis via ERK, Akt and CREB signaling pathways in B16 melanoma cells
Source: Oncotarget. 2017 May 23;8(34):56868–79. doi: 10.18632/oncotarget.18097 (PMC5593609; doi:10.18632/oncotarget.18097)
Supplement: Supplementary file 1 [file oncotarget-08-56868-s001.pdf]

Oleoylethanolamide inhibits α-melanocyte stimulating hormone-stimulated melanogenesis via ERK, Akt and CREB signaling pathways in B16 melanoma cells

Supplementary Figures

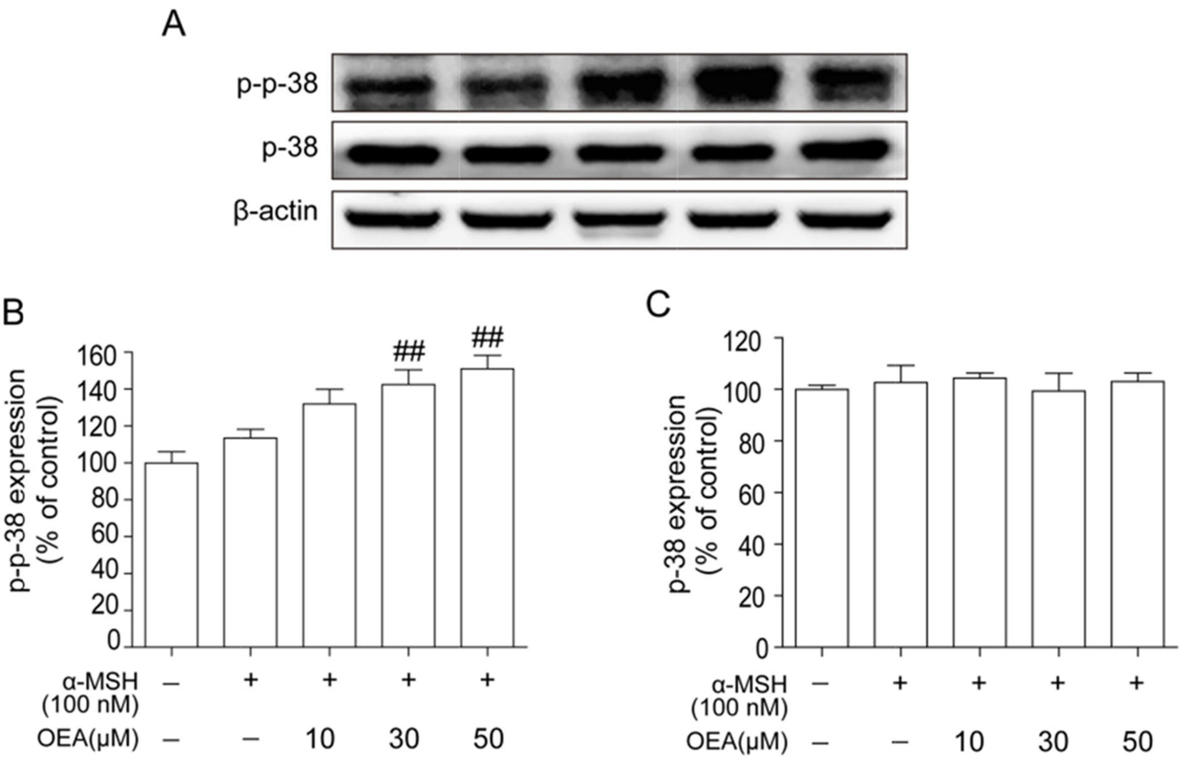

Supplementary Figure 1: Effects of OEA on the activation of the p38 MAPK pathway in α-MSH-stimulated B16 melanoma cells. Cells were exposed to 100 nM α-MSH in the presence of 10, 30, 50 μM OEA. **(A)** p38 and p-p38 protein levels were examined by Western blot. **(B)** The data are presented as percentages compared with the control group (set to 100%) and represented as the means ± SEM of three separate experiments performed in duplicate (n=3). ##*P*<0.01 vs. control group.

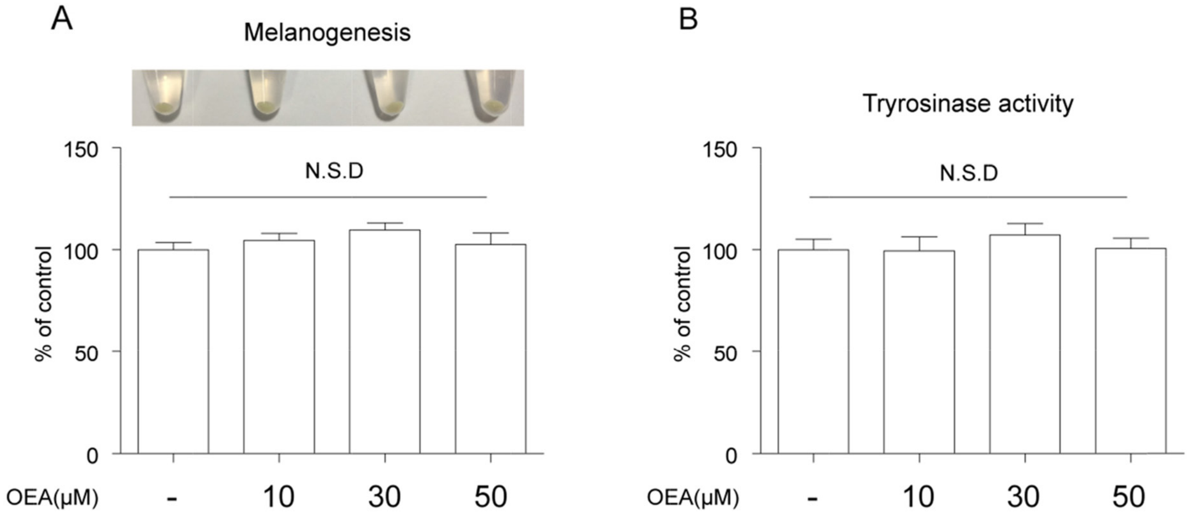

Supplementary Figure 2: Effect of OEA on cellular melanin synthesis and tyrosinase activity in B16 cells. Cells were treated with various concentrations of OEA for 72 h. Relative cellular melanin content **(A)** and tyrosinase activity **(B)** were measured at 72 h after treatment. The percentage values of the treated cells are expressed relative to that in control cells. Data are reported as the mean ± SEM of three independent experiments performed in triplicate (n=3). N.S.D: no significant difference.
